# The Fe-Cyclam-Derived Compound [Fe(cyclam)sal]PF_6_ Restrains Drug-Resistant *Staphylococcus aureus* Proliferation and Biofilm Formation

**DOI:** 10.1021/acsomega.4c11347

**Published:** 2025-03-11

**Authors:** Matheus T. Branca, Thiago P. Silva, Ari S. O. Lemos, Lara M. Campos, Thalita F. Souza, Cinthia Palazzi, Verônica S. Oliveira, Elaine S. Coimbra, Francisco O. N. Silva, Ana Cristina F B Pontes, Ana Carolina M Apolônio, Rossana C. N. Melo, Daniel de L Pontes, Rodrigo L. Fabri

**Affiliations:** † Laboratory of Bioactive Natural Products, Department of Biochemistry, Institute of Biological Sciences, Federal University of Juiz de Fora, Campus, Juiz de Fora, Minas Gerais 36036-900, Brazil; ‡ Laboratory of Cellular Biology, Department of Biology, Institute of Biological Sciences, Federal University of Juiz de Fora, Campus, Juiz de Fora, Minas Gerais 36036-900, Brazil; § Department of Pharmacy, Health Sciences Center, Federal University of Rio Grande do Norte, Campus, Natal, Rio Grande do Norte 59078-970, Brazil; ∥ Laboratory of Parasitology, Department of Parasitology, Microbiology and Immunology, Institute of Biological Sciences, Federal University of Juiz de Fora, Juiz de Fora 36036-900, Brazil; ⊥ Laboratory of Coordination Chemistry and Polymers, Institute of Chemistry, Federal University of Rio Grande do Norte, Natal 59078-970, Brazil; # Laboratory of Bacterial Physiology and Molecular Genetics, Department of Parasitology, Microbiology and Immunology, Institute of Biological Sciences, Federal University of Juiz de Fora, Juiz de Fora 36036-900, Brazil

## Abstract

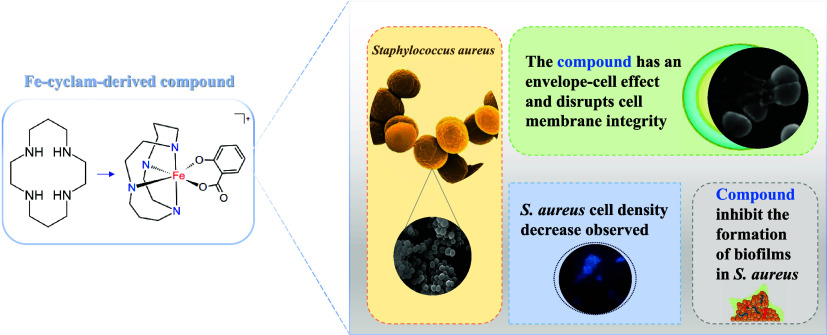

*Staphylococcus aureus* is a bacterium found on the skin and mucous membranes of humans and animals. This micro-organism is classified as an opportunistic pathogen and causes infections in both hospital and community settings. The increase in antibiotic resistance, especially methicillin-resistant *S. aureus* (MRSA), is a major challenge for clinical and epidemiological practice. The present study aims to investigate the potential antibacterial and antibiofilm activities of the compound [Fe(cyclam)sal]PF6 against drug-resistant strains of *S. aureus*. The minimum inhibitory concentration (MIC) and the minimum bactericidal concentration (MBC) against *S. aureus* strains ATCC 25904, *S. aureus* ATCC 33591, and *S. aureus* 05–0052 were determined for [Fe(cyclam)sal]PF_6_. First, bacterial abundance, viability, and cell envelope damage in planktonic cultures were investigated in response to this compound. Second, its potential effect on biofilm proliferation and adhesion was evaluated using different approaches: optical density (OD), scanning electron microscopy (SEM), and biochemical analysis of the extracellular polymeric matrix. The complex [Fe(cyclam)sal]PF_6_ inhibited bacterial growth and induced an increase in cell death. The compound disrupted the integrity of the cell membrane, resulting in the release of cytoplasmic contents into the extracellular medium. Remarkably, the metal complex reduced the pre-established *S. aureus* biofilm and impaired its adhesion. Furthermore, it is not toxic to mammalian cells. The compound [Fe(cyclam)sal]PF_6_ affects both the proliferation and biofilm formation of drug-resistant strains of *S. aureus*, demonstrating strong potential for the design of novel antimicrobial agents.

## Introduction

Antimicrobial resistance has emerged as a major public health crisis in the 21st century, primarily due to the rise of drug-resistant bacterial strains worldwide.^1^ Antibiotic-resistant bacterial infections are estimated to result in over one million deaths annually, posing a significant threat to global public health.^2^ As a key strategy to reduce bacterial resistance, the exploration and development of new therapeutic antimicrobial agents is essential.

*Staphylococcus aureus* is a commensal bacterium that commonly colonizes humans and is considered one of the predominant opportunistic bacterial pathogens.^3^ It causes a diverse spectrum of infections, ranging from minor skin and soft tissue infections to severe and potentially life-threatening conditions such as bacteremia, endocarditis, and pneumonia.^4^ Antibiotic resistance is frequently observed in *S. aureus* isolates, with methicillin-resistant *S. aureus* (MRSA) being the strain most associated with mortality.^2^ MRSA infections are also resistant to other commonly used antibiotics such as penicillin, amoxicillin, and oxacillin. Moreover, nonspecific antibiotic resistance through the formation of biofilms plays a key role in many *S. aureus* biofilm-associated infections.^5,6^

Coordination compounds, i.e., metal complexes with a central metal atom bound to ligands through coordinate covalent bonds, have increasingly been investigated as antibacterial agents with different degrees of antimicrobial activity (reviewed in).^7^ Specifically, metal complexes using cyclam (1,4,8,11-tetraazacyclotetradecane) as a ligand, a molecule with remarkable coordination properties to metal ions, have shown significant potential as antibiotics.^8−12,7,13^

**Figure 1:**
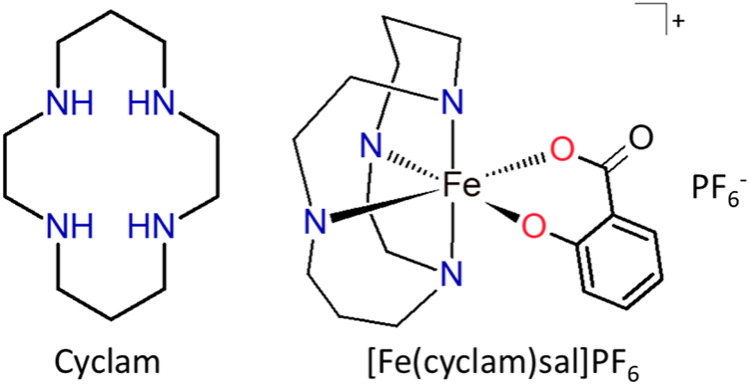
Cyclam ligand (left) and the proposed structure for [Fe(cyclam)sal]PF_6_ (right). Hydrogen atoms were omitted for clarity.

Our group has been developing cyclam-based iron complexes and testing their possible antimicrobial effects against different species of bacteria.^14^ Here, we report on a novel Fe-cyclam complex—[Fe(cyclam)sal]PF_6_—whose proposed structure is presented in Figure 1, in which sal represents a salicylate ion, and its potential biological activity targeting two MRSA strains in both planktonic and biofilm forms.

## Results

### Spectroscopic Characterization

The metal complex was characterized by spectroscopic and electrochemical techniques. The infrared spectrum of [Fe(cyclam)sal]PF_6_ showed vibrational modes characteristic of the presence of the cyclam in the iron(III) coordination sphere, such as the N–H stretching (ν) vibrational modes at 3269 and 3167 cm^–1^, νC–H bands at 2935 and 2878 cm^–1^, as well 1460 cm^–1^, corresponding to the CH_2_ deformation (δ). Furthermore, the spectrum showed bands related to the salicylate ion coordinated to metal, mainly the intense asymmetric (ν_as_OCO) and symmetric (ν_s_OCO) vibrational modes of the carboxylic group, observed respectively at 1602 and 1359 cm^–1^ (Figure S1, Supporting Information). The presence of the PF_6_^–^ counterion is also confirmed by the bands of νPF_6_ at 834 cm^–1^ and angular deformation, δPF_6_, at 555 cm^–1^.

Infrared spectroscopy provides important results for understanding and confirming the coordination of carboxylate ligands to metal centers. These ligands are characterized by evaluating the ν_s_(OCO) and ν_as_(OCO) stretching of the carboxylic group. Particularly, the numerical difference (Δ) between these bands Δ = (ν_as_(OCO) – ν_s_(OCO)) is used as a reference to characterize the coordination arrangement when compared to the uncoordinated carboxylate ion.^15,16^

In complexes where the metal is coordinated to only one of the carboxyl oxygen atoms, the Δ*v*alues are greater than the value found for the uncoordinated ligand. However, when the metal is coordinated bidentate to both oxygen atoms, the Δ*i*s significantly greater than the value of the free ion. Additionally, in binuclear complexes where one metal is bonded to each carboxyl oxygen, the Δvalues are similar to that of the free ion.

The Δfound for uncoordinated salicylate ion is 219 cm^–1^ (for sodium salicylate ν_as_(COO) = 1596 cm^–1^ and ν_as_(COO) = 1377 cm^–1^), Figure S1. For the complex [Fe(cyclam)sal]PF_6_ the Δ_salicylate_ was equal to 243 cm^–1^, suggesting the monodentate coordination mode for the carboxylic group. Additionally, the vibrational mode corresponding to δ(O–H) of the sodium salicylate phenolic group, observed at 1317 cm^–1^ is not present in the spectrum of the complex, thus evidencing the deprotonation of this group due to coordination to the metal center.^17^

These results indicate that the metal is coordinated simultaneously via oxygen atoms of the hydroxyl group and to one of the oxygens of the carboxylate group of salicylate ligand as shown in Figure 1.

**Figure 2:**
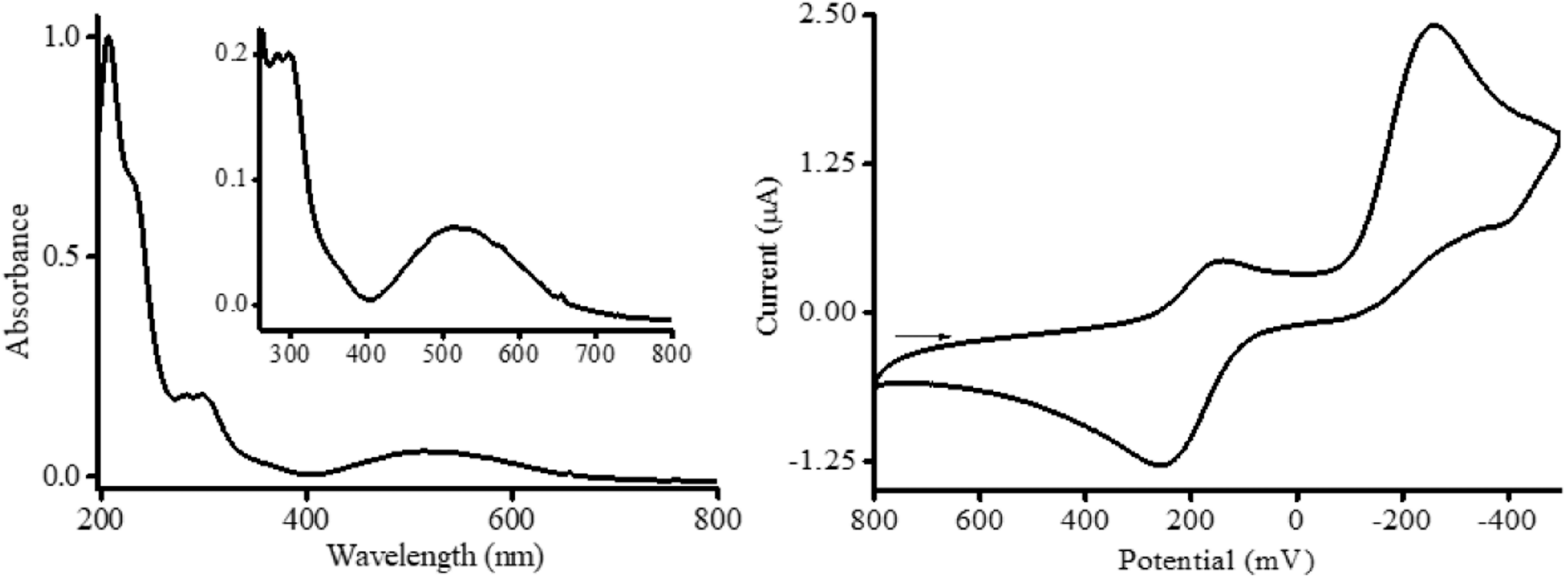
Electronic spectrum (left) in water and cyclic voltammograms in NaTFA 0.1 mol L^–1^, pH = 3.5 at ν = 100 mV s^–1^ of [Fe(cyclam)sal]PF_6_ (right).

The electronic spectrum recorded in water, Figure 2, presented four bands on the ultraviolet region at 207 nm (ε = 2.5 × 10^4^ L mol^–1^ cm^–1^), 229 nm (ε = 1.7 × 10^4^ L mol^–1^ cm^–1^), 282 nm (ε = 4.6 × 10^3^ L mol^–1^ cm^–1^) and 298 nm (ε = 4.6 × 10^3^ L mol^–1^ cm^–1^), referring to intraligand (IL) transitions of salicylate ligand. Particularly the band at 229 nm may also have a cyclam contribution, since the precursor *cis*-[Fe(cyclam)Cl_2_]Cl also has an intraligand band in a similar region, 230 nm (ε = 5.5 × 10^3^ L mol^–1^ cm^–1^). Additionally, the spectra of the complex [Fe(cyclam)sal]PF_6_ presented bands at 361 nm (ε = 7.8 × 10^2^ L mol^–1^ cm^–1^) and 520 nm (ε = 1.4 × 10^3^ L mol^–1^ cm^–1^) referring to LMCT transitions from salicylate ligand to iron(III), pπ sal→ dπ*Fe^3+^.

### Electrochemical Characterization

Cyclic voltammetry of [Fe(cyclam)sal]PF_6_, Figure 2, displays a well-defined Fe^3+/2+^ redox process with half-wave formal potential (*E*_1/2_) of 203 mV vs Ag|AgCl and an irreversible cathodic peak at −256 mV referring to the reduction of the ion salicylate coordinated to the metal.

The electrochemical showed a high sensitivity of the redox potential of iron-cyclam complexes to the nature of additional ligands. The replacement of two *cis-*arranged chlorides in the precursor, *cis*-[Fe(cyclam)Cl_2_]Cl (*E*_1/2_ = 279 mV, *E*_cp_ = 194 mV), by a salicylate ion in the complex [Fe(cyclam)sal]PF_6_ (*E*_1/2_ = 203 mV mV), promoted a substantial decrease of 76 mV in reduction potential of Fe^3+/2+^. This shift indicates that the salicylate ligand promotes a higher stabilization of the metal center in its oxidized state, Fe(III), than chloride ligands.

The window of accessible redox potential in biological systems can vary from −600 to +610 mV vs Ag|AgCl, where the strongest reducing agent in cells corresponds to the nicotinamide adenine dinucleotide phosphate (NADP^+^ + 2e^–^ + H^+^ → NADPH) at −580 mV.^18^ On the other hand, the strongest oxidizing agent is oxygen (O_2_ + 4H^+^ + 4e^–^ → 2H_2_O) at +610 mV, pH 7.0. Interestingly, the iron(III) redox potential found for the compound is within that range, indicating it could be reduced and oxidized in biological systems which can lead to the formation of radical oxygen species or facilitate their interaction with biomolecules.^19^

### MIC and MBC Values for [Fe(cyclam)sal]PF_6_ Compound Indicate a Bacteriostatic Effect against *S. aureus* Strains

First, we demonstrated that the [Fe(cyclam)sal]PF_6_ compound has a clear antibacterial activity with a bacteriostatic effect (MIC = 12.50 μg/mL) detected against both *S. aureus* reference strains (*S. aureus* ATCC 25904 and *S. aureus* ATCC 33591) and clinical isolate (*S. aureus* 05–0052) (Table 1). In contrast, other clinically relevant strains from other bacterial groups (*Enterococcus faecalis* ATCC 19433, *Enterococcus faecium* ATCC 6569, *Streptococcus pneumoniae* ATCC 11733 (MIC = 100.00 μg/mL), and for *Escherichia coli* ATCC 10536, *Klebsiella pneumoniae* ATCC 700603, and *Pseudomonas aeruginosa* ATCC 9027), did not show the same effect (MIC = 200.00 μg/mL). These strains have important clinical relevance and exhibit different resistance characteristics, allowing a comprehensive evaluation of the antibacterial activity of the tested compound. Thus, based on the MIC assay, the most pronounced antibacterial activity was observed against *S. aureus* strains, including both methicillin-resistant (MRSA) and susceptible strains, which were evaluated using the following approaches.

**Table 1:** Minimum Inhibitory Concentration (MIC) and Minimum Bactericidal Concentration (MBC) of [Fe(cyclam)sal]PF_6_ Compound and Amoxicillin against *S. aureus* Strains

	[Fe(cyclam)sal]PF_6_		Amoxicillin
	MIC (μg/mL)	MBC (μg/mL)	MIC (μg/mL)
*S. aureus* ATCC 25904	12.50	>200.00	0.14
*S. aureus* ATCC 33591	12.50	>200.00	12.50
*S. aureus* 05–0052	12.50	>200.00	25.00
*E. faecalis* ATCC 19433	100	>200.00	–
*E. faecium* ATCC 6569	100	>200.00	–
*S. pneumoniae* ATCC 11733	100	>200.00	–
*E. coli* ATCC 10536	200	>200.00	–
*K. pneumoniae* ATCC 700603	200	>200.00	–
*P. aeruginosa* ATCC 9027	200	>200.00	–

MIC values for amoxicillin against all *S. aureus* strains (MIC = 0.14–25.00 μg/mL) indicate that these strains are susceptible to this antibiotic.^20^ MBC values for [Fe(cyclam)sal]PF_6_ were >200 μg/mL. The MBC for amoxicillin could not be determined at the concentrations evaluated.

### [Fe(cyclam)sal]PF_6_ Leads to Bacterial Cell Death

**Figure 3:**
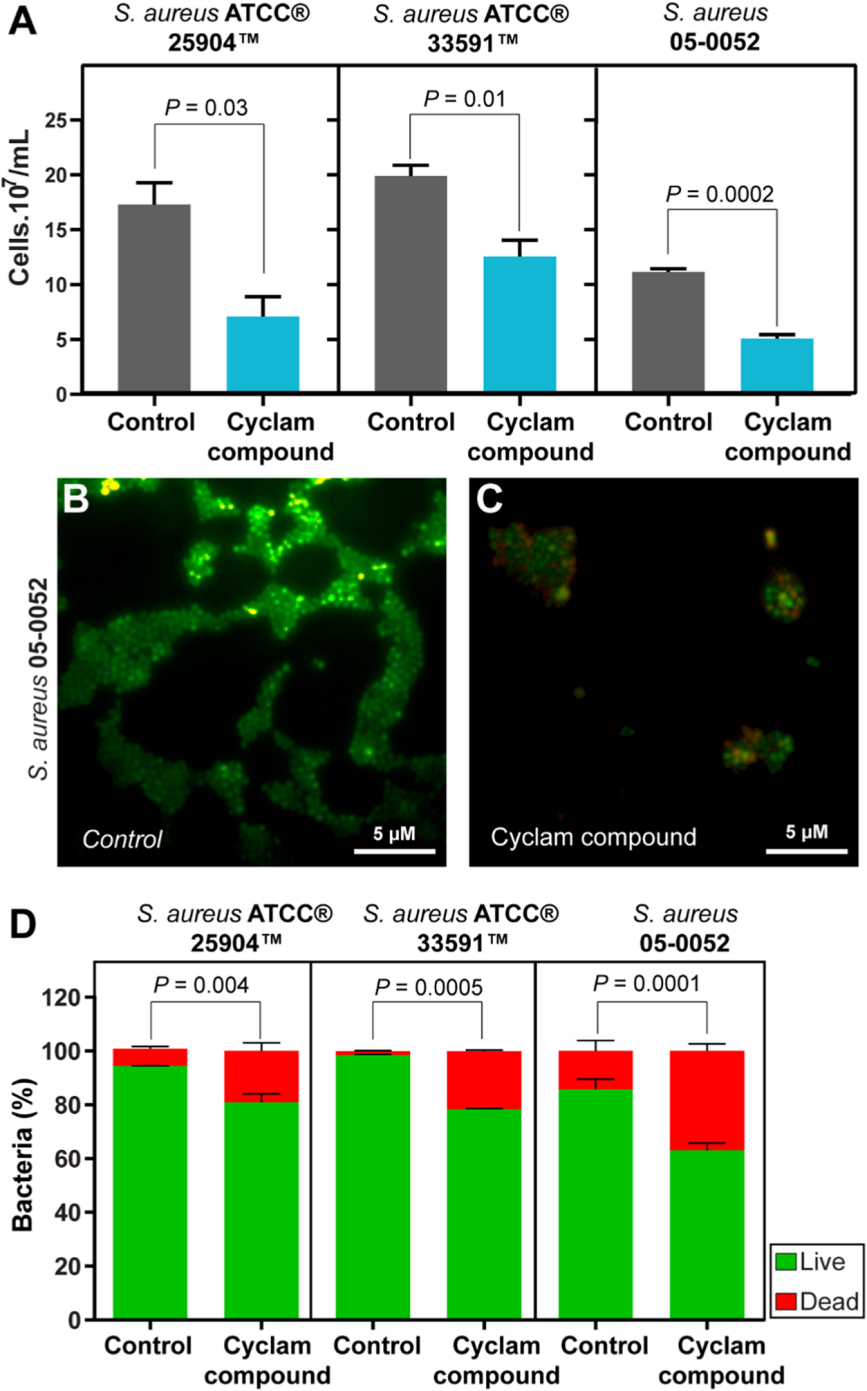
Bacterial cell density and viability of *S. aureus* strains treated with [Fe(cyclam)sal]PF_6_ compound. Cultured bacterial cells were stained with DAPI and quantitated under fluorescence microscopy for cell density analysis (A). Bacterial cells were stained with the LIVE/DEAD BacLight kit, analyzed, and counted under fluorescence microscopy. Live cells are visualized green, while dead bacterial cells are red (B, C). (D) Quantification of live and dead cells in cultures of *S. aureus* ATCC 25904, *S. aureus* ATCC 33591, and *S. aureus* 05–0052 treated or not with [Fe(cyclam)sal]PF_6_. Results are expressed as means ± SEM *P* as indicated by two-way ANOVA test followed by the Bonferroni test.

Next, application of fluorescence microscopy after DAPI staining showed that [Fe(cyclam)sal]PF_6_ affected bacterial density, reducing bacterial growth by 59, 37, and 54% for *S. aureus* ATCC 25904, *S. aureus* ATCC 33591 and *S. aureus* 05–0052, respectively (Figure 3A), compared to the control group.

Backlight staining revealed the presence of live (green) and dead (red) bacteria in all [Fe(cyclam)sal]PF_6_-treated and control groups (Figure 3B,C). Quantitative results showed that the cyclam compound increased the proportion of dead cells in all *S. aureus* cultures when compared to controls (Figure 3D), with the greatest cell death proportion (23%) observed with the *S. aureus* clinical isolate (*S. aureus* 05–0052) (Figure 3D).

### [Fe(cyclam)sal]PF_6_ Affects Bacterial Cell Envelope in Planktonic Cultures

**Figure 4:**
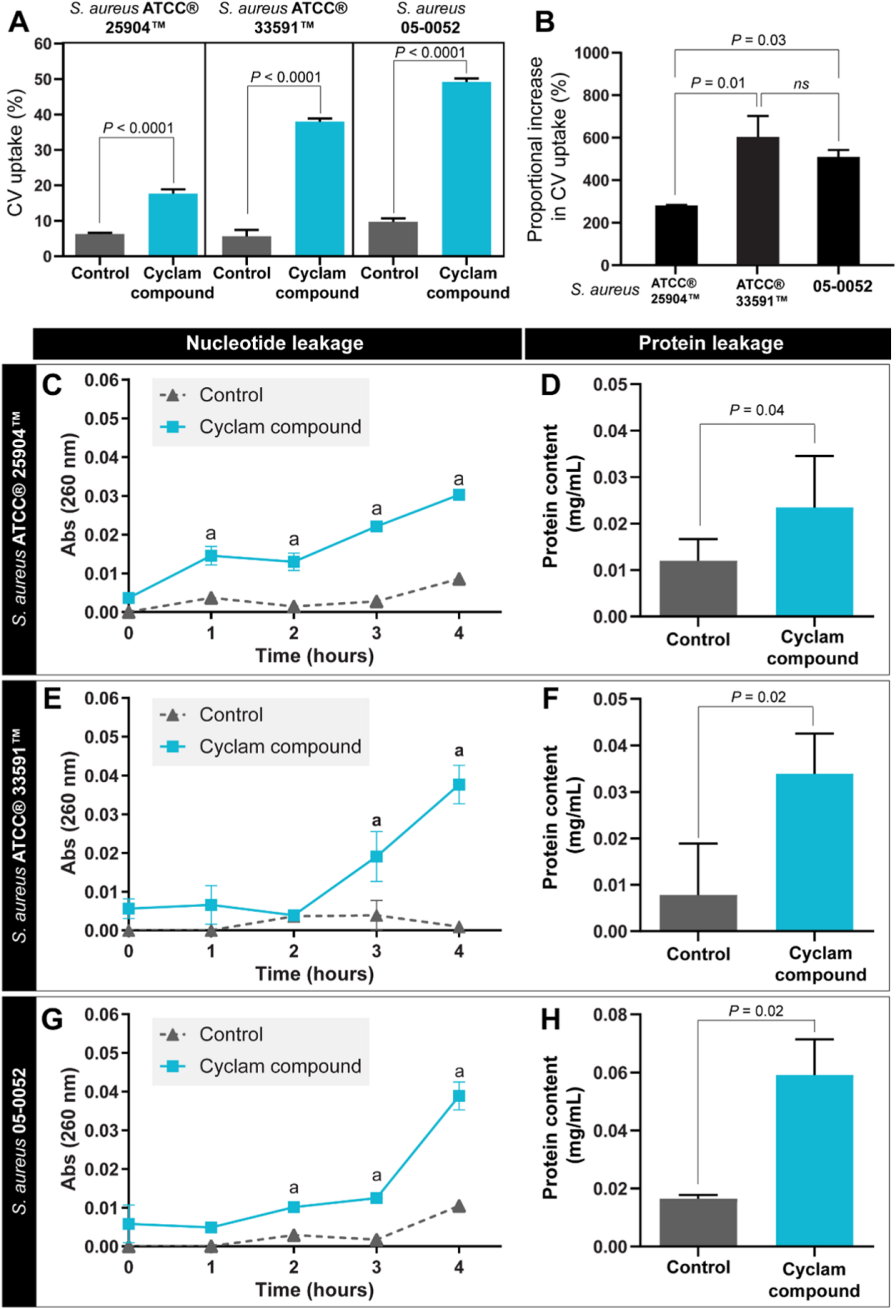
Crystal violet (CV) uptake and release of intracellular contents by [Fe(cyclam)sal]PF_6_-treated *S. aureus* strains. (A) Proportion of CV uptake and (B) proportional increase in CV uptake after [Fe(cyclam)sal]PF6 treatment. (C, E, and G) Extravasation curves of nucleotides into the medium, measured by optical density at 260 nm along 4 h. (D, F, and H) Protein content after 4 h of treatment with [Fe(cyclam)sal]PF_6_ compound. Results are expressed as means ± SEM *P* as indicated by one-way ANOVA test followed by Bonferroni test (A, B) or unpaired *t* test (D, F and H). ^a^ Indicate statistical differences (two-way ANOVA test followed by Bonferroni test, *P* < 0.05).

To investigate how the cyclam compound inhibits bacterial proliferation and nucleotide leakage, crystal violet (CV) and protein uptake assays were applied to identify potential damages in the bacterial envelope.^21^ We found an increase in the bacterial cell permeability in all *S. aureus* strains as evidenced by the increase in CV uptake, when compared with their respective growth controls (Figure 4A). The multidrug-resistant bacterial strains (*S. aureus* ATCC 33591 and *S. aureus* 05–0052) presented the highest increases in CV uptake in comparison with the amoxicillin-susceptible strain ATCC 25904 (P < 0.001; Figure 4A). After treatment with [Fe(cyclam)sal]PF_6_ compound, the CV uptake in relation to the control increased 281.0 ± 2.72, 600.1 ± 99.92, and 510.1 ± 31.97% (mean ± SEM) for *S. aureus* ATCC 25904, *S. aureus* ATCC 33591, and *S. aureus* 05–0052, respectively (Figure 4B).

The cyclam compound also led to an increase in the leakage of the bacterial intracellular components (nucleotide and protein) (Figure 4C–H). The release of nucleotides occurred along 4 h of treatment, mainly after 3 and 4 h of treatment (Figure 4C,E, and G). The protein leakage was higher in [Fe(cyclam)sal]PF_6_ treated cultures for all *S. aureus* strains at 4 h of treatment (Figure 4D,F, and H). The increase in the release of intracellular components indicates that [Fe(cyclam)sal]PF_6_ led to extensive cell envelope damage in all tested *S. aureus* strains.

### [Fe(cyclam)sal]PF_6_ Disrupts Biofilm Adhesion and Proliferation

**Figure 5:**
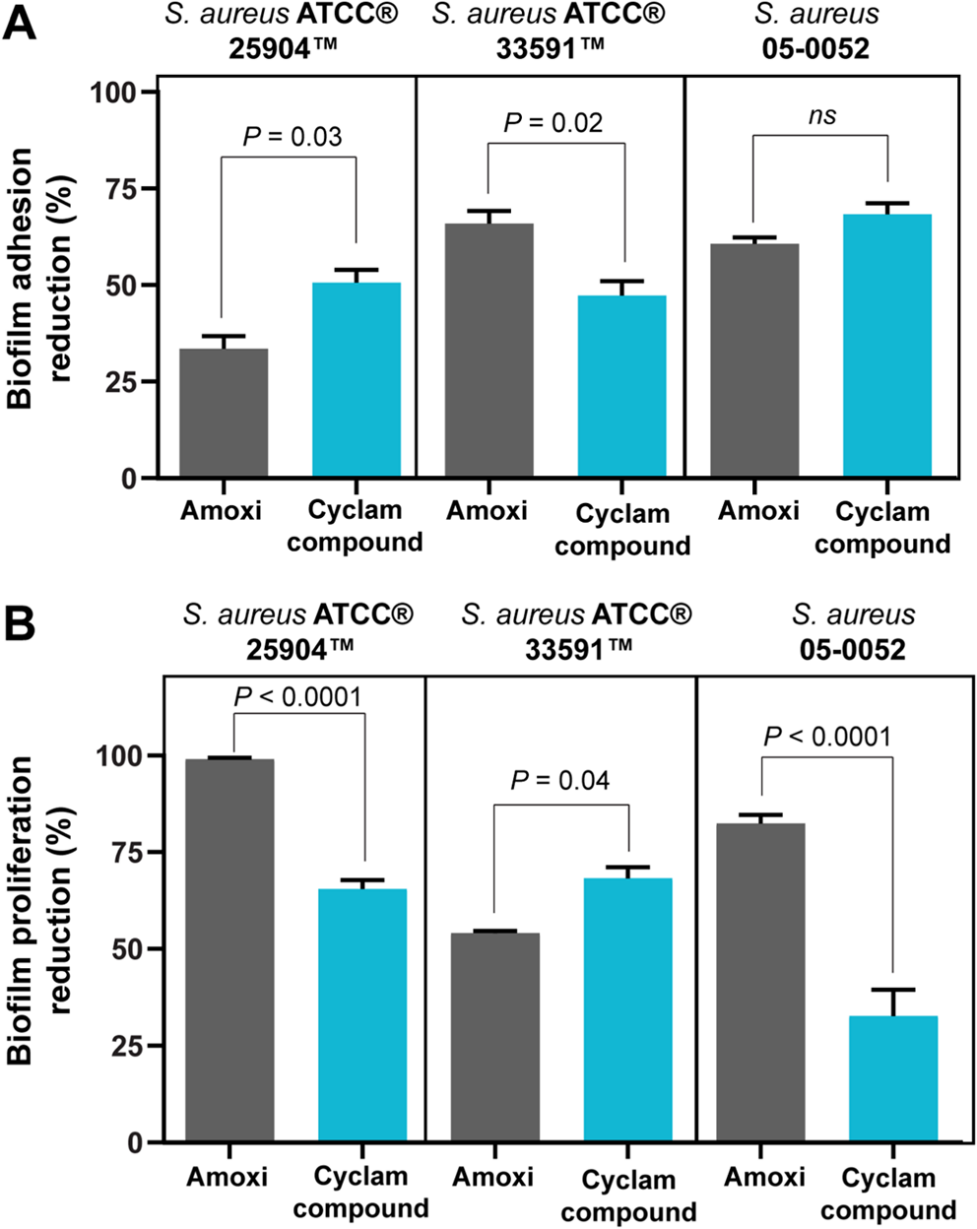
Effects of [Fe(cyclam)sal]PF_6_ and amoxicillin (Amox) on the adhesion (A) and proliferation (B) of *S. aureus* biofilms. Biofilms treated with [Fe(cyclam)sal]PF_6_ or amoxicillin were evaluated according to the percentage of inhibition in relation to growth controls. Results are expressed as means ± SEM *P* as indicated by two-way ANOVA test followed by Bonferroni test.

We next examined the impact of [Fe(cyclam)sal]PF_6_ on the adhesion phase of *S. aureus* biofilm, which characterizes the onset of bacterial biofilm formation.^22^ Spectrophotometric analyses showed that, in all *S. aureus* cultures, both [Fe(cyclam)sal]PF_6_ and amoxicillin treatments inhibited biofilm adhesion (Figure 5A). Compared to the amoxicillin effect on the biofilm adhesion of *S. aureus* ATCC 25904, the iron-cyclam compound had yet a higher inhibitory effect (50.65 ± 6.31% for the complex versus 33.58 ± 3.20% for amoxicillin, mean ± SEM). The *S. aureus* clinical isolate treated with [Fe(cyclam)sal]PF_6_ compound did not present a significant difference in biofilm inhibition in comparison with the amoxicillin treatment (*P* > 0.99; Figure 5A).

Next, we investigated the effect of the [Fe(cyclam)sal]PF_6_ compound on preformed biofilms (Figure 5B). Our analyses showed that both [Fe(cyclam)sal]PF_6_ and amoxicillin treatments led to a significant reduction of the preformed biofilms (Figure 5B). Amoxicillin inhibited almost 100% of growing *S. aureus* ATCC 25904 (an amoxicillin-susceptible strain). The inhibition of biofilms treated with the [Fe(cyclam)sal]PF_6_ compound was 65.47 ± 2.30, 68.27 ± 2.92, 32,63 ± 6.84 (means ± SEM), for *S. aureus* ATCC 25904, *S. aureus* ATCC 33591, and *S. aureus* 05–0052, respectively (Figure 5B). The most accentuated reduction induced by [Fe(cyclam)sal]PF_6_ on biofilm proliferation was noted when the clinical isolate *S. aureus* (*S. aureus* 05–0052) was treated with this compound (Figure 5B).

### Scanning Electron Microscopy (SEM)

**Figure 6:**
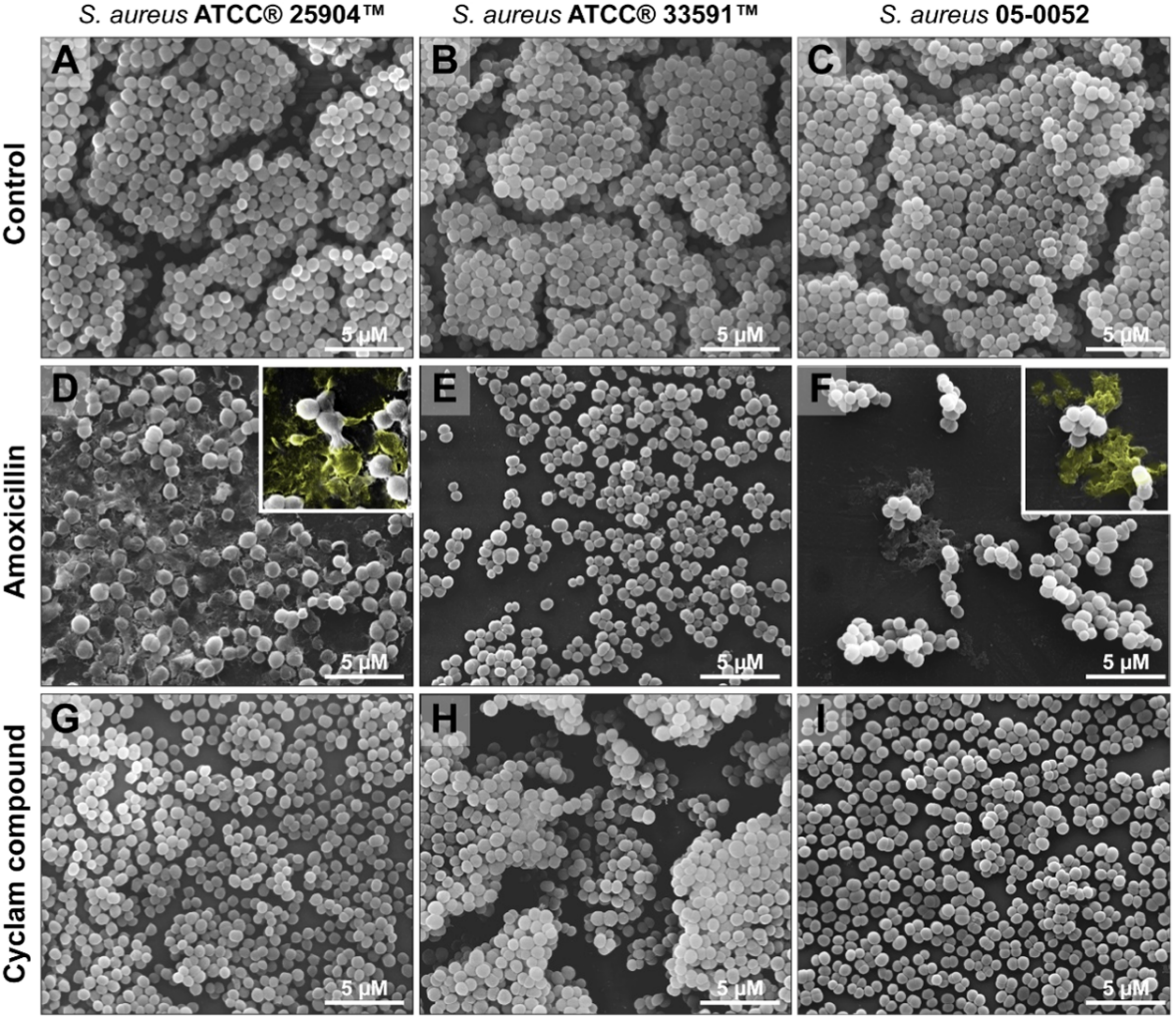
SEM showing biofilms of different bacterial strains normally growing (A–C) or after treatment with amoxicillin (D–F) or [Fe(cyclam)sal]PF_6_ (G–I). Note in high magnification (inset), the structural aspect of biofilms with the presence of amorphous material (A, C, pseudocolored in yellow). Both amoxicillin and [Fe(cyclam)sal]PF_6_ disrupt and reduce bacterial layers, with amoxicillin leading to a more severe effect. Coverslips with biofilms were fixed and processed for SEM.

SEM is a powerful technique to analyze the structural aspects of bacterial biofilms.^23,21^ SEM revealed that all *S. aureus* strains formed highly developed biofilms with a dense population of bacterial cells distributed in several superposed layers (Figure 6A–C). These layers were extensively disrupted after treatment with amoxicillin (Figure 6D–E), with deposition of amorphous material (pseudocolored yellow in Figures 6D,F), likely due to the increase in cell debris from damaged or dead bacteria (Figure 6D,F). When the biofilms of *S. aureus* ATCC 25904 and *S. aureus* 05–0052 were treated with [Fe(cyclam)sal]PF_6_, they showed fewer bacterial layers compared to the controls, thus revealing a disruptive action on their bacterial density, but this effect was less drastic compared to that induced by amoxicillin (Figure 6G–I). Cell bacterial damage was absent or at negligible numbers in the *S. aureus* ATCC 33591 [Fe(cyclam)sal]PF_6_ treated group. Collectively, our findings show that the [Fe(cyclam)sal]PF_6_ compound affects biofilm development by inhibiting bacterial growth.

### [Fe(cyclam)sal]PF_6_ Compound Interferes with the Biofilm Matrix Composition

The biochemical composition of biofilms formed by different *S. aureus* strains was measured by the content of carbohydrates and proteins in the extracellular polymeric matrix (Table 2). Our analysis found that the [Fe(cyclam)sal]PF_6_ and amoxicillin directly interfere with the synthesis of carbohydrates in *S. aureus* 05–0052 biofilm and proteins in *S. aureus* ATCC 25904 biofilm (Table 2).

**Table 2:** Biochemical Composition of Biofilms Formed by *S. aureus* ATCC 25904, *S. aureus* ATCC 33591, and *S. aureus* 05-0052 Treated with [Fe(cyclam)sal]PF_6_ Compound and Amoxicillin

	control		Amoxicillin		[Fe(cyclam)sal]PF_6_	
	TC (μg/mL)	TP (μg/mL)	TC (μg/mL)	TP (μg/mL)	TC (μg/mL)	TP (μg/mL)
*S. aureus* ATCC 25904	26.34 ± 1.50	1.38 ± 0.07	26.88 ± 3.29	1.36 ± 0.005^a^	31.21 ± 4.66	1.51 ± 0.01^a^
*S. aureus* ATCC 33591	26.63 ± 3.12	1.40 ± 0.07	25.16 ± 2.62	1.29 ± 0.08	32.06 ± 4.16	1.43 ± 0.04
*S. aureus* 05–0052	25.72 ± 4.44	1.39 ± 0.10	25.41 ± 3.37^a^	0.63 ± 0.65	46.07 ± 6.54^a^	1.24 ± 0.24

a Indicates statistical difference when compared to the respective controls (ANOVA followed by Bonferroni test, *p* < 0.05). The experiments were performed in triplicate and data represent the mean ± SD **TC:** total carbohydrate and **TP:** total protein.

### Evaluation of Cell Viability in Macrophages

The cytotoxicity of the compound was assessed by cell viability on two macrophage strains, peritoneal macrophages from BALB/c mice and J774A.1 cells (Figure S2). Considering the percentage of cell viability of 70% or more,^24^ all tested concentrations were noncytotoxic (*P* < 0.05).

## Discussion

The investigation of metal complexes has been considered a relevant strategy for the search for novel antimicrobials against resistant bacteria.^12,25^ In the present study, we synthesized and characterized a cationic metal-based complex consisting of iron(III) coordinatively bound to the tetradentate cyclam ligand and to the bidentate salicylate ion (sal), originating the octahedral complex [Fe(cyclam)sal]PF_6_. We demonstrated that this novel compound shows antibiotic activities against two MRSA strains (*S. aureus* ATCC 33591 and *S. aureus* 05–0052) and one susceptible *S. aureus* strain (*S. aureus* ATCC 25904), both in planktonic cultures and biofilms. By combing different approaches, we found that the treatment with [Fe(cyclam)sal]PF_6_: (i) restrains the growth of *S. aureus* strains in planktonic cultures; (ii) affects bacterial cell envelope, and (iii) reduces both biofilm adhesion and proliferation. Our quantitative microscopic analyses using fluorescent markers for cell viability showed that [Fe(cyclam)sal]PF_6_ restrains bacterial growth in planktonic cultures by reducing bacterial cell density and leading to cell death, with the highest level of cell death detected on the MRSA clinical isolate (*S. aureus* 05–0052). Due to the association with the transition metal iron, the cyclam molecule likely contributes to facilitating the entry of the metal into bacteria.^26^ Thus, the compound can interact with biomolecules of the bacterial cell membranes, nucleic acids, nucleic proteins, or other cellular biomolecules leading to structural and functional changes, and eventually to cell death.^27,26^

Our findings demonstrated that the [Fe(cyclam)sal]PF_6_ treatment not only restrained bacterial proliferation but also induced damage to the bacterial envelope. The spectrophotometric data showed that the MRSA strains presented the highest increase in CV uptake after treatment with the iron compound, thus denoting the occurrence of bacterial permeabilization and cell membrane damage.^23,21^ Moreover, we found an increased extravasation of intracellular contents (i.e., nucleotides [low molecular weight] and proteins [macromolecules]) after treatment, especially in the amoxicillin-susceptible strain ATCC 25904. Of note, we detected a gradual release of the bacterial intracellular contents into the extracellular medium with higher compound effectiveness after 3 and 4 h of treatment, indicating significant disruption of the bacterial envelope.^28,21^

A noteworthy finding from the current study was the efficacy of [Fe(cyclam)sal]PF_6_ in both decreasing biofilm adhesion and disrupting preformed biofilms. The initial stage of biofilm formation involves microbial adhesion to an implant surface or a tissue, which enhances survival rate under various stressful conditions.^29,30^ After developing mature biofilms, planktonic bacterial cells disperse, attaching to new surfaces, and starting a new life cycle.^31^ Our spectrophotometric and SEM analyses showed a reduction of the *S. aureus* biofilm extent, an effect detected on a [Fe(cyclam)sal]PF_6_ compound-treated MRSA (05–0052) likely due to bacterial growth inhibition. In addition, our analyses revealed that [Fe(cyclam)sal]PF_6_ can affect carbohydrate and protein biofilm matrix composition. The exopolysaccharide matrix (EPS) comprises most of the biomass of a biofilm and is an essential environment for the maintenance of the bacterial population.^32^ Therefore, our findings support the current view of metal complexes with macrocyclic ligands, such as cyclam, which has increasingly been associated with antibiofilm activities against drug-resistant or susceptible strains in all phases of biofilm establishment and dispersion for several species of bacteria, including *S. aureus*.^31^ It is well documented that positively charged metal complexes can interact with negatively charged bacterial membranes through electrostatic interactions.^33^ The presence of iron in the compound increases its solubility and facilitates interactions with biomolecules such as DNA and proteins.^11^ Additionally, metal-based compounds can disrupt plasma membrane function and enzyme activities through ligand exchange, formation of reactive oxygen species, and depletion of essential substrates.^11^ In addition, the salicylate ligand in our compound contains one oxygen atom from the carbonyl group that can interact with bacterial proteins through hydrogen bonds, resulting in enzyme inhibition.^34,30^ These properties suggest that the [Fe(cyclam)sal]PF_6_ compound, here characterized and tested for the first time, has promised antimicrobial activity acting directly on the bacterial cell envelope disruption.

Therefore, cytotoxicity assays were performed on peritoneal macrophages from BALB/c mice and J774A.1 cells. Assuming a survival rate of 70% or more,^24^ it was possible to verify that for peritoneal macrophages of BALB/c mice and J774A.1 cells, compound [Fe(cyclam)sal]PF_6_ showed no cytotoxic potential, making it a target for further investigation and even new drug design.

Iron complexes have shown remarkable antibacterial potential compared to other metals. For example, Baecker et al.,^35^ evaluated the activity of coordination compounds of Ni(II), Cu(II), Zn(II), Mn(III), Fe(II) and Fe(III) with tetradentate Schiff bases as ligands against *S. aureus* (ATCC 2921 and a methicillin-resistant clinical isolate), *E. coli* (ATCC 25922) and *P. aeruginosa* (ATCC 27853). The results showed that none of the compounds inhibited the growth of Gram-negative bacteria at concentrations below 100 μg/mL and that only iron(III) and manganese(III) compounds were active against *S. aureus*. Five of the seven iron complexes evaluated showed significant inhibition of bacterial growth, with MIC values ranging from 0.781 to 50 μg/mL, depending on the ligand structure. The MIC values found were the same for both *S. aureus* strains tested, and ferroptosis was suggested as part of the mechanism of action.

In another study, Abeydeera et al.,^36^ evaluated the activity of the complex [Fe^III^(hinok)_3_], where hinok is the naturally occurring metal chelator and antimicrobial agent hinokitiol, against both methicillin-sensitive *S. aureus* (MSSA, ATCC 6538) and MRSA (ATCC BAA-44) strains. The results showed that the iron complex was significantly more active against the MSSA strain, with a MIC value of 1.65 μg/mL, than the noncoordinated hinokitiol, which had a MIC value of 9.00 μg/mL. A similar correlation was observed in the MRSA tests, resulting in an MIC value of 3.50 μg/mL for [Fe^III^(hinok)_3_].

## Conclusions

This study found that the [Fe(cyclam)sal]PF_6_ compound has antibacterial activity against *S. aureus* strains ATCC 25904, *S. aureus* ATCC 33591, and *S. aureus* 05–0052. The antibacterial activity includes a bacteriostatic effect with a reduction in bacterial cell density and is mechanistically linked to disruption of the cell envelope. The compound also reduces the preformed biofilm, interfering with its adhesion and composition, and shows no cytotoxicity to mammalian cells. These findings suggest that this metal complex could be valuable for developing drugs to address bacterial resistance and public health initiatives.

## Materials and Methods

### Synthesis of the Complex [Fe(cyclam)sal]PF_6_

The compound was obtained by dissolving the precursor *cis*-[Fe(cyclam)Cl_2_]Cl^37,38^ (172.4 mg, 0.48 mmol) and sodium salicylate (66.2 mg, 0.42 mmol) in 20 mL of distilled water. The reaction mixture was stirred for 5 h at 50 °C and a saturated aqueous solution of NH_4_PF_6_ was added to the reaction. The purple solid obtained was filtered, washed with cold methanol, and dried in a vacuum. Yield: 169.58 mg (66.25%).

IR: ν_max_/cm^–1^; ν(N–H): 3269, 3167; ν(C–H): 2935, 2878; (KBr disk). UV–Vis: λ_max_(H_2_O)/nm (ε/L mol^–1^ cm^–1^): 207 (25,000), 229 (17,000), 282 (4,600), 298 (4,600), 361 (780), 520 (1,400). Elemental analysis (%): calc. for FeC_17_H_29_O_3_N_4_PF_6_: C, 38.00; H, 5.21; N, 10.43%. Found: C, 37.70; H, 5.38; N, 10.70%.

### Spectroscopic and Electrochemistry Characterization

Electronic spectra (UV–Vis) were recorded in solution (water and organic solvents) at room temperature on a photodiode-array spectrophotometer, model 8453 (Agilent). Infrared spectra (400–4000 cm–1) of the compounds dispersed in KBr were recorded on a Shimadzu FTIR-8400S spectrometer. Electrochemical analysis was performed on an Epsilon potentiostat (BASi - Bioanalytical Systems Inc.). Cyclic voltammetric experiments were done in a three-electrode cell. The working electrode was a glassy carbon, a platinum single-wire electrode was used as the counter electrode and an Ag|AgCl electrode saturated with KCl (3.5 mol L^–1^) was used as the reference electrode. The cyclic voltammograms were recorded in sodium trifluoroacetate aqueous solution (NaTFA) 0.1 mol L^–1^, pH = 3.5 at ν = 100 mV s^–1^. Oxygen was removed by purging the solutions with argon. All measurements were performed at 25.0 ± 0.2 °C.

### Bacterial Strains

Two MRSA (*S. aureus* ATCC 33591 and a clinical isolate of *S. aureus* 05–0052) and an amoxicillin-susceptible *S. aureus* ATCC 25904 and reference strains of *E. faecalis* ATCC 19433, *E. faecium* ATCC 6569, *S. pneumoniae* ATCC 11733, *E. coli* ATCC 10536, *K. pneumoniae* ATCC 700603, and *P.aeruginosa* ATCC 9027 were provided by the National Institute for Quality Control in Health at Oswaldo Cruz Foundation (Fiocruz, Rio de Janeiro, Brazil). Prior to testing, all bacterial strains were cultivated on Mueller Hinton Agar (MHA) at 37 °C for 24 h.

### Determination of the Minimum Inhibitory Concentration (MIC)

The antibacterial activity assay was performed using the serial microdilution method.^20^ The [Fe(cyclam)sal]PF_6_ compound was prepared at 500 μg/mL in DMSO 1% and successively diluted to obtain concentrations from 200 to 1.56 μg/mL. Then, 80 μL of the 500 μg/mL stock solution was transferred to microplates containing 100 μL of Mueller–Hinton broth (MHB). To complete the final volume, 20 μL of the bacterial inoculum at 10^6^ CFU/mL (turbidity standard 0.5 on the McFarland scale) was added to the sample contained in each of the wells. Plates were incubated at 37 °C for 24 h. Bacterial growth was monitored using MHB and the complex-free bacterial inoculum and a negative control. Amoxicillin was used as a positive control. All analyses were performed in duplicate in three independent experiments.

### Minimum Bactericidal Concentration (MBC)

The minimum bactericidal concentration (MBC) was determined according to,^39^ with some modifications. After the MIC values were determined, a 10 μL aliquot of the sample was removed from the microplate where there was no visible bacterial growth and then cultured on plates with Mueller-Hinton agar (MHA). The plates were then incubated at 37 °C for 24 h for evaluation.

### Bacterial Density

Bacterial cell quantification was determined by fluorescence microscopy after staining with 4′,6-diamidino-2-phenylindole (DAPI) according.^40^*S. aureus* strains in saline solution were inoculated into MHB containing the [Fe(cyclam)sal]PF_6_ compound (MIC value) and incubated at 37 °C for 24 h. Bacterial strains inoculated in MHB alone served as growth controls. Samples were fixed in 4% formaldehyde, stained with 0.1 μg/mL DAPI, and cytocentrifuged (Shandon cytospin 4, Thermo Electron) at 452 g for 10 min at high acceleration. Bacterial cells on the slides were counted under a fluorescence microscope (BX-60, Olympus, Melville, NY, USA) in 20 random fields at 1,000× magnification. The total number of bacteria was determined by multiplication with the dilution factor. Experiments and analyses were performed in triplicate.

### Bacterial Viability

Bacterial cell viability was assessed by using the LIVE/DEAD BacLight Kit (Molecular Probes), which enables differentiation between live and dead bacteria according to their membrane integrity.^40^ Precultured *S. aureus* strains were prepared in sterile saline (10^8^ CFU/mL) and then inoculated into tubes with MHB containing the [Fe(cyclam)sal]PF_6_ (MIC value) to a final concentration of 10^6^ CFU/mL. The tubes were then incubated for 24 h at 37 °C. An aliquot of 1 mL of the bacterial cells in suspension was stained with the reagents and incubated under light for 30 min. Samples were prepared by cytocentrifugation at 500g for 10 min at high acceleration.^39^ The resulting slides were analyzed under a fluorescence microscope (BX-60, Olympus, Melville, NY). A total of 10 random fields per slide were analyzed and the proportion of live/dead bacterial cells was determined. All experiments and analyses were performed in triplicate.

### Crystal Violet Permeability

The crystal violet (CV) assay is used to identify potential changes in bacterial membrane permeability since this dye readily penetrates bacterial cells with compromised membranes.^41,42^*S. aureus* strains were incubated in MHB with [Fe(cyclam)sal]PF_6_ (MIC value) for 4 h at 37 °C, as.^21^ After centrifugation, the pellets were resuspended in CV solution (10 μg/mL in sterile water) for 10 min at 35 °C, centrifuged again and OD measured in the supernatant at 570 nm using a spectrophotometer (Multiskan Go, Thermo Scientific, Waltham, MA). The untreated cell group was used as a growth control. The OD value of the crystal violet solution was taken as 100%. The percentage of CV uptake was expressed as follows: [(OD value of sample/OD value of CV solution) × 100].^21^

### Nucleotide Leakage and Total Protein Dosage

Fresh cultures of *S. aureus* were prepared as cell suspensions (10^6^ CFU/mL PBS, 10 mM, pH 7.4) and incubated with the [Fe(cyclam)sal]PF_6_ compound at its MIC value for 1, 2, 3, and 4 h. After incubation and subsequent centrifugation, nucleotide leakage in the supernatants was assessed by measuring the optical density (OD) at 260 nm.^21^ Cultures without the compound served as controls.

The supernatant (at 4 h) was reserved for total protein dosage, which was performed as before.^43^ First, 10 μL of the supernatant was aliquoted into test tubes containing water and 2 mL of reactive solution (potassium sodium tartrate 2%, copper sulfate 1%, and alkaline solution of NaOH and Na_2_CO_3_). After homogenization, the samples were allowed to stand for 10 min. After this period, 2 mL of Follin’s solution was added. The tubes were homogenized again and allowed to stand for another 30 min. An aliquot from each tube was then added to a 96-well plate and read at 660 nm in a spectrophotometer (Multiskan Go, Thermo Scientific, Waltham, MA) and the absorbances were compared with an albumin calibration curve. All experiments were conducted in triplicate.

### Effect on Biofilm Adhesion

The biofilm adhesion is the first step to the establishment of biofilms, where single bacterial cells begin to adhere to a substratum.^22^ The biofilm assay was performed according to.^44^ Briefly, 100 μL of *S. aureus* cell suspensions (2 × 10^8^ CFU/mL) were incubated with [Fe(cyclam)sal]PF_6_ (MIC value) at 37 °C for 24 h, in 96-well microtiter plates. Loose bacteria cells were gently removed by washing each well with 200 μL of PBS. Then, the adhered cells were stained with 0.1% crystal violet at room temperature for 30 min. The wells were washed with PBS and biofilms were fixed with 200 mL of 96% ethanol for 15 min. Adhered biofilms were analyzed by spectrophotometry (570 nm). The assay was done in triplicate. Reduction in biofilm adhesion was calculated as follows: [OD (control) – OD (treatment)/OD (control)] × 100.

### Effect on Biofilm Proliferation

The [Fe(cyclam)sal]PF_6_ (MIC value) was tested on already-formed biofilms.^23^ Biofilms of the bacterial strains without planktonic cells were incubated with the compound and the OD was measured at 600 nm before (time 0 h) and after (time 24 h) incubation (37 °C/24 h). Inoculum in the MHB alone was used as growth control. The biofilm inhibition was calculated by the proportion of reduction considering the growth control as 100% of OD. Thus, the percentage of biofilm inhibition was obtained by the equation: [(OD (growth control) – OD (treatment))/OD (growth control)] × 100. The experiment was conducted in triplicate.

### Scanning Electron Microscopy (SEM)

*S. aureus* strains were seeded on MHA, incubated for 24 h at 37 °C, and inoculated into a tube containing 5 mL of MHB supplemented with 1% glucose. Then, 500 μL of the inoculated broth (2 × 10^8^ CFU/mL) was added to 24-well plates containing round glass coverslips (13 mm, Glasscyto). The treatment (*n* = 3 wells) was done by adding 500 μL of the [Fe(cyclam)sal]PF_6_ compound (MIC). For negative (*n* = 3) and positive (*n* = 3) controls 500 μL of sterile water or amoxicillin (MIC) were added, respectively. Biofilms formed on glass coverslips were incubated for 24 h at 37 °C and then fixed in 2.5% glutaraldehyde in 0.1 M cacodylate buffer for 30 min at room temperature.^23^ Next, biofilms on glass coverslips were postfixed with osmium tetroxide and dehydrated through a graded series of ethanol solutions (30, 50, 70, 90%, and twice in 100%) for 15 min at each concentration. Coverslips were mounted on aluminum holders, sputtered with 5 nm gold, and analyzed in a scanning electron microscope (JEOL JSM-6390LV, Tokyo, Japan) for observation of the biofilms and bacterial morphology.

### Biochemical Composition of the Extracellular Polymeric Matrix

The biochemical composition of the extracellular matrix was analyzed according to,^44^ with modifications. Biofilms were disrupted by sonication in an ultrasonic water bath for 5 min. The cell suspensions were then pooled and centrifuged (3,000 rpm for 10 min). The supernatant was used as a source to study the biochemical composition of the extracellular matrix using carbohydrate (CHO) dosage by the phenol-sulfuric acid method,^45^ and protein dosage by the Lowry method.^43^

### Cytotoxicity Assays

Macrophages obtained from the peritoneal cavity of BALB/c mice, previously inoculated with 3% thioglycolate medium, were used for cell viability assays. Macrophages were initially cultured in microtiter plates at a concentration of 2 × 10^5^ cells per well in RPMI-1640 medium supplemented with 2 mM l-glutamine (5% SFB and 100 μg/mL penicillin/streptomycin) maintained at 37 °C in a 5% CO_2_ atmosphere. Different concentrations of [Fe(cyclam)sal]PF_6_ compound were then added in triplicate (18.75 to 300 μg/mL) and the final volume made up to 100 μL. The cells were then incubated for 48 h in an incubator at 37 °C under a 5% CO_2_ atmosphere and the MTT (3-(4,5-dimethylthiazol-2-yl)-2,5-diphenyltetrazolium bromide) test was performed. Dimethyl sulfoxide (DMSO) at 0.06% was used as a negative control. The protocol for the use of animals was approved by the Animal Research Ethics Committee of the Federal University of Juiz de Fora (UFJF), under (n° 007/2018-CEUA). For J774A.1 cells the same culture procedure was performed, but at a concentration of 1 × 10^5^ cells per well.

### Evaluation of Cell Viability by MTT Assay

The MTT colorimetric method was used to determine cell viability after exposure to the compound [Fe(cyclam)sal]PF_6_. The method is based on the metabolism of the MTT salt by cells, which is reduced within the mitochondria to a product called formazan. Once solubilized, the product formed (formazan) can be quantified and its colorimetric intensity is directly proportional to the number of viable cells.^46^ After the incubation period, 10 μL of MTT dye (5 mg/mL) was added by completing the well volume with RPMI-1640 medium supplemented to 100 μL, followed by incubation at 37 °C with 5% CO_2_ atmosphere for 2 h and 30 min to form formazan crystals. At the end, the medium was removed and 100 μL DMSO was added to each well to solubilize the crystals. After color stabilization, the plates were read at a wavelength of 595 nm.

### Statistical Analysis

Data were expressed using parametric analysis of variance (one- or two-away ANOVA) followed by Bonferroni post-test or means (unpaired test t). GraphPad Prism 8 software (La Jolla CA) was used to perform the analyses. All data were expressed as mean and standard deviation, with a significance level of 5% (*p* < 0.05).

## Supporting Information Available

Infrared spectra of [Fe(cyclam)sal]PF6 and sodium salicylate (Figure S1). Cytotoxicity against peritoneal macrophages from BALB/c mice and J774A.1 of [Fe(cyclam)sal]PF6 (Figure S2) (PDF)

## Abbreviations

Cyclam: 1,4,8,11-tetraazacyclotetradecane
MRSA: methicillin-resistant *Staphylococcus aureus*
MIC: minimum inhibitory concentration
NaTFA: sodium trifluoroacetate
DAPI: 4′,6-diamidino-2-phenylindole
PBS: buffered saline
DMSO: dimethyl sulfoxide
CV: crystal violet uptake
OD: optical density
SEM: scanning electron microscopy
EPS: exopolysaccharide matrix
MTT: (3-(4,5-dimethylthiazol-2-yl)-2,5-diphenyltetrazolium bromide)

## References

[ref1] Chin K. W.; Tiong H. L. M.; Luang-In V.; Ma N. L. An overview of antibiotic and antibiotic resistance. Environ. Adv. 2023, 11, 100331 10.1016/j.envadv.2022.100331.

[ref2] Murray C. J. L.; Ikuta K. S.; Sharara F.; et al. Global burden of bacterial antimicrobial resistance in 2019: a systematic analysis. Lancet 2022, 399, 629–655. 10.1016/S0140-6736(21)02724-0. 35065702 PMC8841637

[ref3] Lowy F. D. *Staphylococcus aureus* infections. N. Engl. J. Med. 1998, 339, 520–532. 10.1056/NEJM199808203390806. 9709046

[ref4] Howden B. P.; Giulieri S. G.; Wong F. L. T.; Baines S. L.; Sharkey L. K.; Lee J. Y. H.; Hachani A.; Monk I. R.; Stinear T. P. *Staphylococcus aureus* host interactions and adaptation. Nat. Rev. Microbiol. 2023, 21, 380–395. 10.1038/s41579-023-00852-y. 36707725 PMC9882747

[ref5] Otto M. Staphylococcal Biofilms. Microbiol. Spectrum 2018, 6 (4), 207–228. 10.1128/microbiolspec.GPP3-0023-2018. PMC6282163 30117414

[ref6] Idrees M.; Sawant S.; Karodia N.; Rahman A. *Staphylococcus aureus* biofilm: Morphology, genetics, pathogenesis and treatment strategies. Int. J. Environ. Res. 2021, 18, 7602 10.3390/ijerph18147602. PMC8304105 34300053

[ref7] Ke H.; Hu F.; Meng L.; Chen Q. H.; Lai Q. S.; Li Z. C.; Huang Z. L.; Liao J. Z.; Qiu J. D.; Lu C. Z. Ultrastable radical-doped coordination compounds with antimicrobial activity against antibiotic-resistant bacteria. Chem. Commun. 2020, 56, 14353–14356. 10.1039/D0CC06379G. 33169746

[ref8] Grabchev I.; Yordanova S.; Bosh P.; Vasileva-Tonkova E.; Kukeva R.; Stoyanov S.; Stoyanova R. Structural characterization of 1,8-naphthalimides and *in vitro* microbiological activity of their Cu (II) and Zn (II) complexes. J. Mol. Struct. 2017, 1130, 974–983. 10.1016/j.molstruc.2016.10.073.

[ref9] Alves L. G.; Portel J. F.; Sousa A. S.; Ferreira O.; Almada S.; Silva E. R.; Martins A. M.; Leitão J. H. Investigations into the Structure/Antibacterial Activity Relationships of Cyclam and Cyclen Derivatives. Antibiotics 2019, 8, 224 10.3390/antibiotics8040224. 31739454 PMC6963676

[ref10] Abdel-Rahman L. H.; Abdelhamid A. A.; Abu-Dief A. M.; Shehata M. R.; Bakheet M. A. Facile synthesis, X-Ray structure of new multi-substituted aryl imidazole ligand, biological screening and DNA binding of its Cr(III), Fe(III) and Cu(II) coordination compounds as potential antibiotic and anticancer drugs. J. Mol. Struct. 2020, 1200, 127034 10.1016/j.molstruc.2019.127034.

[ref11] Claudel M.; Schwarte J. V.; Fromm K. M. New Antimicrobial Strategies Based on Metal Complexes. Chemistry 2020, 2, 849–899. 10.3390/chemistry2040056.

[ref12] Frei A.; Zuegg J.; Elliott A. G.; Baker M.; Braese S.; Brown C.; Chen F.; Dowson C. G.; Dujardin G.; Jung N.; King A. P.; Monsour A. M.; Massi M.; Moat J.; Mohamed H. A.; Renfrew A. K.; Rutledge P. J.; Sandler P. J.; Todd M. H.; Willans C. E.; Wilson J. J.; Cooper M. A.; Blaskovic M. A. T. Metal complexes as a promising source for new antibiotics. Chem. Sci. 2020, 11, 2627–2639. 10.1039/C9SC06460E. 32206266 PMC7069370

[ref13] Archana B.; Sreedaran S. New cyclam based Zn(II) complexes: effect of flexibility and para substitution on DNA binding, *in vitro* cytotoxic studies and antimicrobial activities. J. Chem. Sci. 2022, 134, 102 10.1007/s12039-022-02091-9.

[ref14] Polo A. B.; Lemos A. S. O.; da Mata C. P. S. M.; Oliveira V. S.; Pontes A. C. F. B.; Pontes D. L.; Tavares G. D.; Fabri R. L.; Apolônio A. C. M. *In vitro* activity of the novel Fe-cyclam complex against clinical multidrug-resistant bacterial isolates from Brazil. Future Microbiol. 2023, 18, 897–909. 10.2217/fmb-2023-0058. 37584550

[ref15] Nakamoto K. Infrared and Raman Spectra of Inorganic and Coordination Compounds: Part B: Applications in Coordination, Organometallic, and Bioinorganic Chemistry 6ath ed.; John Wiley & Sons, Inc., 2009.

[ref16] Porwal S. K.; Furia E.; Harris M. E.; Viswanathan R.; Devireddy L. Synthetic, potentiometric and spectroscopic studies of chelation between Fe(III) and 2,5-DHBA supports salicylate-mode of siderophore binding interactions. J. Inorg. Biochem. 2015, 145, 1–10. 10.1016/j.jinorgbio.2014.12.010. 25589161

[ref17] Philip D.; John A.; Panicker C. Y.; Varghese H. T. FT-Raman, FT-IR and surface enhanced Raman scattering spectra of sodium salicylate. Spectrochim. Acta, Part A 2001, 57, 1561–1566. 10.1016/S1386-1425(01)00395-X. 11471708

[ref18] Jungwirth U.; Kowol C. R.; Keppler B. K.; Hartinger C. G.; Berger W.; P Christian H. Anticancer Activity of Metal Complexes: Involvement of Redox Processes. Antioxid. Redox Signaling 2011, 15, 1085–1127. 10.1089/ars.2010.3663. PMC3371750 21275772

[ref19] de Medeiros W. M. T. Q.; Medeiros M. J. C.; Carvalho E. M.; Lima J. A.; Oliveira V. S.; Pontes A. C. F. B.; Silva F. O. N.; Ellena J. A.; Rocha H. A. O.; Sousa E. H. S.; Pontes D. L. A Vanillin-based copper(II) metal complex with a DNA-mediated apoptotic activity. RSC Adv. 2018, 8, 16873–16886. 10.1039/C8RA03626H. 35540529 PMC9080323

[ref20] CLSI. Methods for Dilution Antimicrobial Susceptibility Tests for Bacteria that Grow Aerobically;Twenty-Eight Informational Supplement: M07, Clinical and Laboratory Standards Institute 2018.

[ref21] Campos L. M.; Lemos A. S. O.; Silva T. P.; Oliveira L. G.; Nascimento A. L. R.; Carvalho J. J.; Moraes A. C. N.; Rocha V. N.; Aguiar J. A. K.; Scio E.; Apolônio A. C. M.; Melo R. C. N.; Fabri R. L. *Mitracarpus frigidus* is active against *Salmonella enterica* species including the biofilm form. Ind. Crops Prod. 2019, 141, 111793 10.1016/j.indcrop.2019.111793.

[ref22] Moormeier D. E.; Bayles K. W. *Staphylococcus aureus* biofilm: a complex developmental organism. Mol. Microbiol. 2017, 104, 365–376. 10.1111/mmi.13634. 28142193 PMC5397344

[ref23] Lemos A. S. O.; Campos L. M.; Melo L.; Guedes M. C. M. R.; Oliveira L. G.; Silva T. P.; Melo R. C. N.; Rocha V. N.; Aguiar J. A. C.; Apolônio A. C. M.; Scio E.; Fabri R. Antibacterial and antibiofilm activities of psychorubrin, a pyranonaphthoquinone isolated from *Mitracarpus frigidus* (Rubiaceae). Front. Microbiol. 2018, 9, 724 10.3389/fmicb.2018.00724. 29706943 PMC5908958

[ref24] I. O. F. Standardization. ISO10993–5: Biological Evaluation of Medical Devices-Part 5: Tests for in vitro Cytotoxicity ISO: Geneva; 2009.

[ref25] Tesauro D. Metal Complexes in Diagnosis and Therapy. Int. J. Mol. Sci. 2022, 23, 4377 10.3390/ijms23084377. 35457194 PMC9024768

[ref26] Evans A.; Kavanagh K. A. Evaluation of metal-based antimicrobial compounds for the treatment of bacterial pathogens. J. Med. Microbiol. 2021, 70, 001363 10.1099/jmm.0.001363. 33961541 PMC8289199

[ref27] Thurman R. B.; Gerba C. P.; Bitton G. The molecular mechanisms of copper and silver ion disinfection of bacteria and viruses. J. Crit. Rev. Environ. Sci. Technol. 1989, 18, 295–315. 10.1080/10643388909388351.

[ref28] Hao G.; Shi Y. H.; Tang Y. L.; Le G. W. The membrane action mechanism of analogs of the antimicrobial peptide Buforin 2. Peptides 2009, 30, 1421–1427. 10.1016/j.peptides.2009.05.016. 19467281

[ref29] Singh R.; Ray P.; Das A.; Sharma M. Penetration of antibiotics through *Staphylococcus aureus* and *Staphylococcus epidermidis* biofilms. J. Antimicrob. Chemother. 2010, 65, 1955–1958. 10.1093/jac/dkq257. 20615927

[ref30] Payandeh J.; Volgraf M. Ligand binding at the protein-lipid interface: strategic considerations for drug design. Nat. Rev. Drug Discovery 2021, 20, 710–722. 10.1038/s41573-021-00240-2. 34257432

[ref31] Olar R.; Badea M.; Chifiriuc M. C. Metal Complexes—A Promising Approach to Target Biofilm Associated Infections. Molecules 2022, 27, 758 10.3390/molecules27030758. 35164021 PMC8838073

[ref32] Nasser A.; Dallal M.; Jahanbakhshi S.; Azimi T.; Nikouei L. *Staphylococcus aureus*: biofilm formation and strategies against it. Curr. Pharm. Biotechnol. 2022, 23, 664–678. 10.2174/1389201022666210708171123. 34238148

[ref33] Kotynia A.; Wiatrak B.; Kamysz W.; Neubauer D.; Jawień P.; Marcianiak A. Cationic Peptides and Their Cu(II) and Ni(II) Complexes: Coordination and Biological Characteristics. Int. J. Mol. Sci. 2021, 22, 12028 10.3390/ijms222112028. 34769458 PMC8584440

[ref34] Bissantz C.; Kuhn B.; Stahl M. A Medicinal Chemist’s Guide to Molecular Interactions. J. Med. Chem. 2010, 53, 5061–5084. 10.1021/jm100112j. 20345171 PMC2905122

[ref35] Baecker D.; Sesli Ö.; Knabl L.; Huber S.; Orth-Höller D.; Gust R. Investigating the antibacterial activity of salen/salophene metal complexes: Induction of ferroptosis as part of the mode of action. Eur. J. Med. Chem. 2021, 209, 112907 10.1016/j.ejmech.2020.112907. 33069056

[ref36] Abeydeera N.; Yu B.; Pant B. D.; Kim M. H.; Huang S. D. Harnessing the toxicity of dysregulated iron uptake for killing *Staphylococcus aureus*: reality or mirage?. Biomater. Sci. 2022, 10 (2), 474–484. 10.1039/D1BM01743H. 34904144 PMC8860634

[ref37] Guilard R.; Siri O.; Tabard A.; Broeker G.; Richard P.; Nurco D. J.; Smith K. M. One-pot synthesis, physicochemical characterization and crystal structures of *cis*- and *trans*-(1,4,8,11- tetraazacyclotetradecane)-dichloroiron(III) complexes. J. Chem. Soc. Dalton Trans. 1997, 19, 3459–3463. 10.1039/a702775c.

[ref38] Sousa E. H. S.; Oliveira C. P.; Vasconcellos L. C. G.; Lopes L. G. F.; Diogenes I. C. N.; Carvalho I. M. M.; Miranda J. C. V.; Dias F. A.; Moreira I. S. Thermal isomerization of *cis*-[Fe(cyclam)Cl_2_]Cl·H_2_O complex in the solid state. Thermochim. Acta 2001, 376, 141–145. 10.1016/S0040-6031(01)00576-7.

[ref39] Spencer J. F. T.; Spencer A. L. R. Public Health Microbiology: Methods and Protocols; Springer Science & Business Media, 2008.

[ref40] Silva T. P.; Noyma N. P.; Duque T. L. A.; Gamalier J. P.; Vidal L. O.; Lobão L. M.; Garcia H. C.; Roland F.; Melo R. C. N. Visualizing aquatic bacteria by light and transmission electron microscopy. Antonie van Leeuwenhoek 2014, 105, 1–14. 10.1007/s10482-013-0047-6. 24132727

[ref41] Devi K. P.; Nisha A. S.; Sakthivel R.; Pandian S. K. Eugenol (an essential oil of clove) acts as an antibacterial agent against *Salmonella typhi* by disrupting the cellular membrane. J. Ethnopharmacol. 2010, 130, 107–115. 10.1016/j.jep.2010.04.025. 20435121

[ref42] Halder S.; Yadav K. K.; Sarkar R.; Mukherjee S.; Saha P.; Haldar S.; Karmakar S.; Sen T. Alteration of Zeta potential and membrane permeability in bacteria: a study with cationic agents. SpringerPlus 2015, 4, 672 10.1186/s40064-015-1476-7. 26558175 PMC4633473

[ref43] Lowry O. H.; Rosebrough N. J.; Farr A. L.; Randall R. J. Protein measurement with the Folin phenol reagent. J. Biol. Chem. 1951, 193, 265–275. 10.1016/S0021-9258(19)52451-6. 14907713

[ref44] Plyuta V.; Zaitseva J.; Lobakova E.; Zagoskina N.; Kuznetsov A.; Khmel I. Effect of plant phenolic compounds on biofilm formation by *Pseudomonas aeruginosa*. APMIS 2013, 121, 1073–1081. 10.1111/apm.12083. 23594262

[ref45] Dubois M.; Gilles K. A.; Hamilton J. K.; Rebers P. T.; Smith F. Colorimetric method for determination of sugars and related substances. Anal. Chem. 1956, 28, 350–356. 10.1021/ac60111a017.

[ref46] Mosmann T. Rapid colorimetric assay for cellular growth and survival: application to proliferation and cytotoxicity assays. J. Immunol. Methods 1983, 65, 55–63. 10.1016/0022-1759(83)90303-4. 6606682

