# Supplementary material for: The Fe-Cyclam-Derived Compound [Fe(cyclam)sal]PF6 Restrains Drug-Resistant Staphylococcus aureus Proliferation and Biofilm Formation
Source: ACS Omega. 2025 Mar 11;10(11):11386–96. doi: 10.1021/acsomega.4c11347 (PMC11947776; doi:10.1021/acsomega.4c11347)

Supporting information

The Fe-Cyclam-derived compound [Fe(cyclam)sal]PF_6 restrains drug-resistant *Staphylococcus aureus* proliferation and biofilm formation

Matheus T. Branca[a]#, Thiago P. Silva[b]#, Ari S. O. Lemos[a], Lara M. Campos[a], Thalita F. Souza[a], Cinthia Palazzi[b], Verônica S. Oliveira[c], Elaine S. Coimbra[d], Francisco O. N. Silva[e], Ana Cristina F. B. Pontes[e], Ana Carolina M. Apolônio[f], Rossana C. N. Melo[b], Daniel de L. Pontes[e], and Rodrigo L. Fabri[a]*

# These authors contributed equally to this work.

[a]*Laboratory of Bioactive Natural Products, Department of Biochemistry, Institute of Biological Sciences, Federal University of Juiz de Fora, Campus, Juiz de Fora, MG, Brazil;*

[b]*Laboratory of Cellular Biology, Department of Biology, Institute of Biological Sciences, Federal University of Juiz de Fora, Campus, Juiz de Fora, MG, Brazil;*

[c]*Department of Pharmacy, Health Sciences Center, Federal University of Rio Grande do Norte, Campus, Natal, RN, Brazil;*

[d]*Laboratory of Parasitology, Department of Parasitology, Microbiology and Immunology, Institute of Biological Sciences, Federal University of Juiz de Fora, Juiz de Fora, Brazil;*

[e]*Laboratory of Coordination Chemistry and Polymers, Institute of Chemistry, Federal University of Rio Grande do Norte, Natal, Brazil;*

[f]*Laboratory of Bacterial Physiology and Molecular Genetics, Department of Parasitology, Microbiology and Immunology, Institute of Biological Sciences, Federal University of Juiz de Fora, Juiz de Fora, Brazil.*

*Corresponding author: E-mail: rodrigo.fabri@ufjf.br.

Figure S1 - Infrared spectra of (a) [Fe(cyclam)sal]PF_6 and (b) sodium salicylate from 1300 to 1650 cm^{-1} emphasizing the differences between the asymmetric (ν_{as}) and symmetric (ν_s) vibrational stretching modes of the carboxylic (OCO) group in the salicylate ion and after its coordination to iron(III).

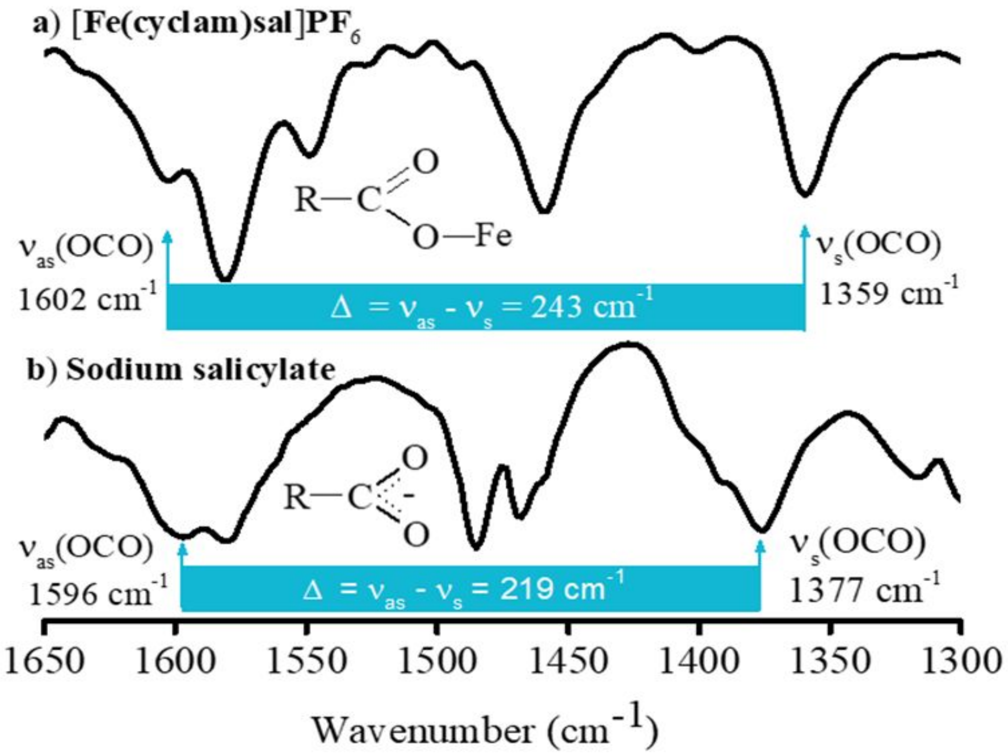

Figure S2 - Cytotoxicity of $[Fe(cyclam)sal]PF_6$ compound against peritoneal macrophages from BALB/c mice (A) and J774A.1 cells (B) after 48 hours of exposure. Macrophages were incubated with different concentrations of the compound (18.75 to 300 µg/mL). The cell viability was determined by the MTT colorimetric method. The result was expressed as the percentage of survival in relation to untreated cells. The red dashed line indicates the cell viability percentage of 70%. The experiments were performed in triplicate and data represent the mean ± SD (ANOVA followed by Bonferroni, $p<0.05$).

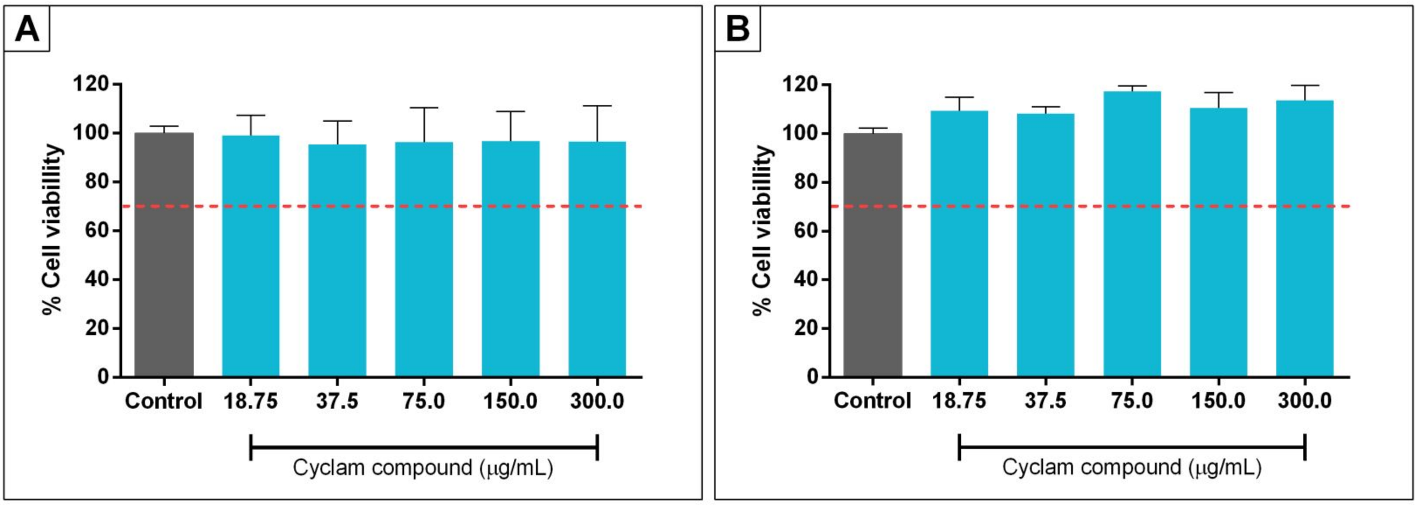

Supplement: Supplementary file 1 — ao4c11347_si_001.pdf [file ao4c11347_si_001.pdf]
